# Fabrication of magnetic manganese ferrite-loaded sugar cane bagasse/peanut peel biochar adsorbents for the adsorptive removal of phosphorus from aqueous solution

**DOI:** 10.1038/s41598-025-08753-1

**Published:** 2025-07-05

**Authors:** Alyaa I. Salim, Nada A. Abdelgawad, Ehab Rozaik, Nagwan G. Mostafa

**Affiliations:** 1 School of Biotechnology, 26th of July Corridor, Nile University, Sheikh Zayed City, 12588 Giza Egypt; 2 Environmental and Sanitary Engineering Division, Public Works Department, Faculty of Engineering, Cairo University, Giza, 12613 Egypt

**Keywords:** Adsorption kinetics, Magnetic nanoparticles loaded BC, Phosphorus removal, Regeneration studies, Thermodynamics, Sustainability, Magnetic properties and materials

## Abstract

Adsorption has the potential to be a highly effective and selective method for recovering and adsorbing phosphate from wastewater and water, which can serve as secondary sources of phosphorus. The objectives of this study were to synthesize manganese ferrite (MF) nanoparticles which are fabricated and studied alone and loaded on sugar cane bagasse and peanut peels biochar (BC) adsorbents (Mn@Fe_3_O_4_@BC) by in-situ growth method, which in turn applied to evaluate their capabilities for phosphorus adsorption from aqueous solutions. Batch experiments were conducted to determine the optimum adsorption conditions for different process parameters such as pH, adsorbent dose, and initial phosphorus concentration. The maximum phosphorous removal efficiency using MF, MFBC_b_, and MFBC_p_ was obtained at adsorbent doses 0.2 and 0.3 g/L, and initial phosphorous concentrations of 20, 40, and 60 mg/L, respectively. The optimum retention time was obtained at 120 min for MF and MFBC_b_, and 150 min for MFBC_p_. The optimum rotation speed and temperature were 120 rpm and 25 °C for all adsorbents. The maximum removal efficiencies obtained are 98.5% and 99% for MF and MFBCs, respectively. Different characterization analyses; including SEM, EDX, and FTIR; were applied to investigate surface morphology, elemental composition, and chemical properties of the adsorbents before and after the adsorption process. Adsorption kinetics, isotherms, capacity, mechanisms, and thermodynamic studies were studied to evaluate the adsorption process. And finally, adsorbents were regenerated using their magnetic properties and a second successive adsorption cycle was evaluated showing promising results for MFBC adsorbents which can affect the expected costs of the adsorption process.

## Introduction

Phosphorus, a key nutrient in aquatic ecosystems, is an essential nutrient for plants’ growth, however, when such nutrient exists in large amounts it causes *eutrophication*^1,2^. Therefore, phosphorus should be kept at acceptable levels to prevent eutrophication while maintaining sufficient conditions for plants and aquatic life growth^3,4^. Eutrophication, a process that leads to algal blooms, which depletes oxygen levels in water bodies (hypoxia) and disrupts aquatic ecosystems^5^. Likewise, harmful algal blooms release toxins that are hazardous to human health^6^. Additionally, oxygen-depleted zones, or “dead zones,” emerge as a result of algal decay, leading to the death of aquatic life^7^. High levels of phosphorus impair water clarity, affecting its usability for drinking, recreation, and industrial purposes.

Phosphorus in wastewater originates from various anthropogenic and natural sources, including agricultural runoff, through fertilizers rich in phosphates, domestic wastewater contains phosphorus from detergents, cleaning agents, and human excreta, industries such as food processing, pharmaceuticals, and chemical manufacturing discharge phosphorus-rich effluents, and finally, urban stormwater often contains phosphorus from soil erosion, organic debris, and road dust^8^. Global water quality standards recommend phosphorus concentrations of less than 0.1 mg/L in surface waters, while untreated wastewater can contain levels exceeding 10 mg/L, highlighting the urgent need for effective phosphorus removal technologies^9^.

There are many approaches used for phosphorus removal such as biological treatment, chemical precipitation, and adsorption processes^10^. From here came the need to develop cheap economic friendly techniques that can achieve high removal efficiency, less quantities of sludge, and higher potential of being applied on a large scale. As a result, the adsorption process emerged which proved high efficiency in phosphorus removal while being simply operated at an acceptable cost^11^. Phosphorus adsorption performance is a critical factor in the development of effective wastewater treatment technologies. Various materials have been studied for their ability to adsorb phosphorus, including activated carbon, known for its high surface area and porosity^12^, biochar, shown promise in phosphorus adsorption due to its porous structure and functional groups^13^, and Magnetic Nanoparticles (MNPs), which offer high adsorption capacities and can be easily separated from solutions using magnetic fields^14^.

In recent years, removal of phosphorus from wastewater by adsorption has attracted much attention^15–17^. Many low cost adsorbents were tested for phosphorus removal; such as fly ash, steel slag, zirconium, active red mud, and iron humate; however, they showed low adsorption capacity^18^. The economic concerns and adsorption capacity are not the only aspects that define the most appropriate adsorbent, the ability of adsorbent to adapt to diverse pH values and existence of other constituents present in wastewater shall be considered as well. So that, it has been resorted to nanotechnology in wastewater treatment.

The nano adsorbents give higher efficiency than other adsorbents because of their small pore size, larger surface area and the absence of internal diffusion resistance and as a consequent they provide better kinetics for adsorption of metal ions from aqueous solutions^19–22^. Many MNPs were used in previous studies for phosphorus removal such as ZnFe_2_O_4_^23^, NiFe_2_O_4_^24^, MgFe_2_O_4_^25^, CoFe_2_O_4_^26^, Fe_3_O_4_^27^, and Fe_2_O_3_^28^. Nanoparticles are considered excellent adsorbents, however, most MNPs agglomerate easily because of their high surface energy. In order to improve the dispersion stability, MnFe_2_O_4_ supported on biochar was promoted because of its positive electric characteristics in addition to strong magnetism, high natural abundance, and low ecotoxicity^15,16,29^.

Phosphorus adsorption could be improved by using magnetic nanoparticle supported on biochar^30,31^. Biochar is black gold in many fields such as environmental treatment, soil amendment and wetland construction because of its high adsorption capacity, low density, and great surface area^32^. It is formed through pyrolysis of biomass under oxygen-free or oxygen-limited conditions. Also it has the ability to improve the treated wastewater characteristics by removing pollutants in addition to being cost-effective^16,33^.

In this study, phosphorus removal from aqueous solutions using MNP supported on biochar is investigated. The manganese ferrite MnFe_2_O_4_ solo (MF) and supported on biochar (MFBCs), which is prepared using sugar cane bagasse and peanut peel agriculture wastes, are applied. The effect of various factors such as initial concentration of phosphorus, dose of adsorbents, pH, retention time, and rotation speed of the shaker; on phosphorus removal efficiency and adsorption capacity are investigated. The optimum conditions for phosphorus removal are obtained for the different considered adsorbents. Also, adsorption mechanism, kinetics, isotherm, and thermodynamics are studied. The adsorbents are analyzed using SEM, EDX, and FTIR tests. Finally, the adsorbents were regenerated after their first use and tested for a second adsorption cycle. The second-cycle removal efficiency for different adsorbents is evaluated.

## Materials and methods

### Materials and instruments

Materials and chemicals used in this study are listed in Table 1. Temperature and pH were measured using a digital multimeter (HI2550 meter, HANNA). The phosphorus concentration was determined via a 6305 spectrophotometer, JENWAY. All chemical solutions were prepared using ultrapure water.

**Table 1:** Chemicals used for Preparation of phosphorous aqueous solutions, nanoparticles and biochar.

Material	Chemical formula	Purity (%)
Ferric Chloride Hexahydrate	FeCl_3_.6H_2_O	97
Potassium Permanganate	KMnO_4_	97–100
Potassium Dihydrogen Orthophosphate	KH_2_PO_4_	98-100.5
Ammonium Molybdate 4H_2_O	((NH_4_)_6_Mo_7_O_24_.4H_2_O, Crystals	98
Ammonium Metavanadate	NH_4_VO_3_	98
Hydrochloric Acid	HCL	30–33%
Sodium Hydroxide (concentration 98%)	NaOH	-
Ethyl alcohol (concentration 96%)	C_2_H_5_OH	-

### Methods

#### Preparation of magnetic nanoparticle MnFe_2_O_4_ (MF)

The MF composite was prepared by co-precipitation method. Firstly, a 0.3 M (8.109 g) of ferric chloride hexahydrate (FeCl_3_.6H_2_O) was added to 100 mL of ultra-pure water. Secondly, another solution of 0.1 M (1.58 g) of potassium permanganate (KMnO_4_) was added to 100 mL of ultra-pure water, Thirdly, both solutions were mixed together with continuous stirring. Fourthly, the final solution was maintained at pH 10 by adding sodium hydroxide (NaOH) dropwise. Finally, the solution was left for an hour to form the suspension; then the suspended matters were filtered and washed well several times with ultra-pure water and ethanol, after that the solution was dried in the oven to remove residual liquids.

#### Preparation of Biochar (BC)

Peanut peel, which was collected from local market in Giza city, Egypt, was washed with tap water; then it was dried in the oven at 100 °C for 2 h to remove moisture and grinded. After that, samples were pyrolyzed in muffle under aerobic conditions at a rate of 10 °C/min up to 500 °C for 3 h at maximum temperature; then it was cooled at room temperature to produce biochar from peanut peel (BC_p_).

Bagasse used in this study was also obtained from local market in Giza city, Egypt was first boiled to git red of any sugar. After that the same steps used for peanut peel were applied for preparing biochar from bagasse (BC_b_).

#### Preparation of nanoparticles MnFe_2_O_4_ Biochar composites (MFBCs)

The MFBC composite was prepared, also, by co-precipitation method. Firstly, a 0.3 M (8.109 g) of ferric chloride hexahydrate (FeCl_3_.6H_2_O) was added to 100 mL of ultra-pure water. Secondly, 1 g of biochar (bagasse and peanut peel each separately) was added to the previous solution. After that, previous steps from 2 till 5 used for preparation of the MF are repeated. The yield of the MFBC composite was calculated as the ratio of the mass of the final dried composite to the total mass of precursors (FeCl₃·6 H₂O + biochar). The typical yield was found to be approximately 72.4%, indicating efficient composite formation under the conditions described.

### Phosphorus batch experiments

A stock solution with phosphorus concentration of 100 ppm was prepared by dissolving potassium dihydrogen phosphate (906 mg/L) in ultra-pure water. The stock solution was then diluted to achieve the required concentration for the batch test. Six batch experiments were conducted to obtain the optimum parameters including the initial concentration of phosphorus (C_0_), the pH, the adsorbent doses of MF and MFBCs, the shaker rotation speed, and the retention time. The initial conditions of adsorption batch experiments were as follows: (1) the initial concentration of phosphorus solution was 20 mg/L, (2) the initial pH of the reaction was 7.0, (3) dosage of MF and MFBCs was 1.0 g/L, (4) rotational speed of the shaker was 120 rpm, (5) the temperature of the reaction was 25 °C, (6) contact time for adsorption experiments was 2 h. The batch experiments were conducted such that one of the parameters was tested and its optimum value obtained while the other parameters were maintained constantly. The optimum value obtained would then be held constant during the following batches. The batch experiments were conducted in the order and tested value ranges summarized in Tables 2 and 3, and 4.

The measurement of phosphorus was performed at 520 nm wavelength using the stannous chloride technique^34^. The basic idea behind the process was that stannous chloride reduces molybdophosphoric acid to brightly colored molybdenum blue. The intensity of the yellow color gradually fading was a sign of declining phosphorus concentration.

**Table 2:** Parameters tested using MF nanoparticles in batch experiments.

Batch #	Selected parameter(s) from previous batches	Tested parameter	Tested values	Optimum value
**1**	-	pH	1, 3, 5, 6, 7, and 10	5–6*
**2**	• pH = 5	MF dose	0.1, 0.2, 0.3, 0.4, 0.6, 0.8, and 1 g/L	0.2 g/L
**3**	• pH = 5 • MF dose = 0.2 g/L	Initial phosphorus concentration (*C*_*0*_)	20, 40, 60, and 80 mg/L	20 mg/L
**4**	• pH = 5 • MF dose = 0.2 g/L • Initial phosphorus concentration (*C*_*0*_) = 20 mg/L	Shaker rotation speed	100, 120, 150, and 200 rpm	120 rpm
**5**	• pH = 5 • MF dose = 0.2 g/L • Initial phosphorus concentration (*C*_*0*_) = 20 mg/L • Shaker rotation speed = 120 rpm	Retention time	30,60, 90, 120,150, and 180 min	120 min
**6**	• pH = 5 • MF dose = 0.2 g/L • Initial phosphorus concentration (*C*_*0*_) = 20 mg/L • Shaker rotation speed = 120 rpm • Retention time = 120 min	Temperature	25 °C, 35 °C, and 45 °C	25 °C

*: optimum range of pH.

**Table 3:** Parameters tested using MFBC_b_ in batch experiments.

Batch #	Selected parameter(s) from previous batches	Tested parameter	Tested values	Optimum value
**1**	-	pH	1, 3, 5, 6, 7, and 10	3–5*
**2**	• pH = 3	MFBC_b_ dose	0.1, 0.2, 0.3, 0.4, 0.6, 0.8, and 1 g/L	0.3 g/L
**3**	• pH = 3 • MFBC_b_ dose = 0.3 g/L	Initial phosphorus concentration (*C*_*0*_)	20, 40, 60, and 80 mg/L	40 mg/L
**4**	• pH = 3 • MFBC_b_ dose = 0.3 g/L • Initial phosphorus concentration (*C*_*0*_) = 40 mg/L	Shaker rotation speed	100, 120, 150, and 200 rpm	120 rpm
**5**	• pH = 3 • MFBC_b_ dose = 0.3 g/L • Initial phosphorus concentration (*C*_*0*_) = 40 mg/L • Shaker rotation speed = 120 rpm	Retention time	30,60, 90, 120,150, and 180 min	120 min
**6**	• pH = 3 • MFBC_b_ dose = 0.3 g/L • Initial phosphorus concentration (*C*_*0*_) = 40 mg/L • Shaker rotation speed = 120 rpm • Retention time = 120 min	Temperature	25 °C, 35 °C, and 45 °C	25 °C

*: optimum range of pH.

**Table 4:** Parameters tested using MFBC_p_ in batch experiments.

Batch #	Selected parameter(s) from previous batches	Tested parameter	Tested values	Optimum value
**1**	-	pH	1, 3, 5, 6, 7, and 10	3–5*
**2**	• pH = 3	MFBC_p_ dose	0.1, 0.2, 0.3, 0.4, 0.6, 0.8, and 1 g/L	0.3 g/L
**3**	• pH = 3 • MFBC_p_ dose = 0.3 g/L	Initial phosphorus concentration (*C*_*0*_)	20, 40, 60, and 80 mg/L	60 mg/L
**4**	• pH = 3 • MFBC_p_ dose = 0.3 g/L • Initial phosphorus concentration (*C*_*0*_) = 60 mg/L	Shaker rotation speed	100, 120, 150, and 200 rpm	120 rpm
**5**	• pH = 3 • MFBC_p_ dose = 0.3 g/L • Initial phosphorus concentration (*C*_*0*_) = 60 mg/L • Shaker rotation speed = 120 rpm	Retention time	30,60, 90, 120,150, and 180 min	150 min
**6**	• pH = 3 • MFBC_p_ dose = 0.3 g/L • Initial phosphorus concentration (*C*_*0*_) = 60 mg/L • Shaker rotation speed = 120 rpm • Retention time = 150 min	Temperature	25 °C, 35 °C, and 45 °C	25 °C

*: optimum range of pH.

### Adsorption studies

The adsorption capacity (*q*_*e*_) and the efficiency of phosphorus removal are calculated using the following equations^35^:

1 $$\:{q}_{e}=\frac{\left({C}_{0}-{C}_{t}\right)*V}{m},$$

2 $$\:Removal\:Efficiency\:\%=\frac{\left({C}_{0}-{C}_{t}\right)}{{C}_{0}}*10,$$

where *q*_*e*_ is the adsorption capacity (mg/g), *C*_*0*_ and *C*_*t*_ are the phosphorus concentrations (mg/L) at times 0 and t, respectively, *V* is the volume of the solution (mL); and *m* is the mass of MF and MFBCs particles (mg).

In batch 3, where the initial phosphorus concentrations (*C*_*0*_) were investigated, the retention time was extended to three hours so as to substitute in adsorption kinetics and isotherm models. The kinetics of phosphorus adsorption on MF and MFBCs were investigated using three models; pseudo-first-order (Eq. (3)), pseudo-second-order (Eq. (4)), and Elovich (Eq. (5)) kinetics models^36^.

3 $$\text{ln}\left({q}_{e}-{q}_{t}\right)=ln{q}_{e}-{K}_{1}*t,$$

4 $$\:\frac{t}{{q}_{t}}=\frac{t}{{q}_{e}}+\frac{1}{{K}_{2}.{{q}_{e}}^{2}},$$

5 $$\:{q}_{t}=\frac{1}{\beta\:}\text{ln\:}\left(\alpha\:\beta\:\right)+\frac{1}{\beta\:}ln\:\left(t\right),$$

where *t* is the contact time (minutes), $\:{q}_{e}$and $\:{q}_{t}$ are adsorption capacities (mg/g) at equilibrium and time t, respectively. *k*_*1*_ is the pseudo-first-order rate constant (min^− 1^) and *K*_*2*_ is the pseudo-second-order rate constant (g/mg.min). *α* (mg/g.min) and *β* (g/mg) are the initial adsorption rate constant and the Elovich adsorption constant, respectively.

The equilibrium studies conducted at different initial phosphorus concentrations were fitted with the linearized Freundlich^37^ (Eq. (6)), Langmuir^38^ (Eq. (7)), Temkin^39^ (Eq. (8)), and Dubinin–Radushkevich (D–R)^10,40^ (Eq. (9)) adsorption isotherm Eq. 

6 $$\:\text{l}\text{o}\text{g}{\:q}_{e}=log{K}_{F}+\frac{1}{n}\text{log}{C}_{e},$$

where *q*_*e*_ is the amount of phosphorus adsorbed (mg/g) at equilibrium, $\:{C}_{e}$ is the equilibrium concentration (mg/L), $\:n$ and *K*_*F*_ are constants incorporating all factors affecting the adsorption process such as intensity and adsorption capacity, respectively.

7 $$\:\frac{{C}_{e}}{{q}_{e}}=\:\frac{1}{{K}_{L}{q}_{m}}+\:\frac{{C}_{e}}{{q}_{m}},$$

where *C*_*e*_ is the equilibrium concentration (mg/L), *q*_*e*_ is the amount of phosphorus adsorbed (mg/g) at equilibrium, *q*_*m*_ is the maximum adsorption capacity (mg/g) and *K*_*L*_ (L/mg) is Langmuir constant related to energy of adsorption.

8 $$\:{q}_{e}=\:\frac{RT}{b}\:ln{K}_{T}+\:\frac{RT}{b}\:ln{C}_{e},$$

where $\:{q}_{e}$ is the amount of phosphorus adsorbed (mg/g) at equilibrium, *K*_*T*_ was the Temkin isotherm constant (L/g), R is the gas constant (8.314 J/mol K); *T* is the temperature of the solution in kelvin (*K*), b is the Temkin constant related to the heat of adsorption (J/mol), $\:{C}_{e}$is the equilibrium concentration (mg/L).

9 $$\:\text{ln}{q}_{e}=ln{q}_{d}-\:\beta\:*{\epsilon\:}^{2},$$

where *q*_*e*_ is the amount of phosphorus adsorbed (mg/g) at equilibrium, *q*_*d*_ is the D-R constant related to the maximum coverage (mg/g), *β* is the constant associated to adsorption energy (mol^2^/J^2^), and *ε* is the Polanyi potential which is equal to *RT ln (1 + 1/C*_*e*_*).*

Adsorbate transfer in solid-liquid adsorption is typically governed by either intra-particle diffusion, external surface diffusion, or both, where adsorption mechanism is divided into three controlling steps; film diffusion, surface adsorption, and intra-particle diffusion. The intra-particle diffusion model^40^, Eq. (10), and Boyd kinetic model^41^, Eq. (11), were used to determine the rate-limiting step that controls the adsorption rate.

10 $$\:{q}_{t}=\:{k}_{i}*{t}^{0.5}+C,$$

where q_t_ is the amount of phosphorus adsorbed at time t (mg/g), k_i_ is the intra-particle diffusion rate constant (mg/g·min^0.5^), and C (mg/g) is a constant that is related to the thickness of the layer.

11 $$\:Bt=\:-0.4977-ln\left(1-\frac{{q}_{t}}{{q}_{e}}\right),$$

where Bt is a mathematical function of q_t_/q_e_. The adsorption mechanism was established through implementation of the intra-particle diffusion model, then he actual rate-limiting step in the adsorption process is defined by the Boyd kinetic model which was used to determine whether surface diffusion or pore diffusion was the slowest step in the adsorption of phosphorus ions.

The adsorption process is significantly affected by temperature, and as a result it is very important to study process thermodynamics. The inherent energetic changes related to the adsorption process were determined by obtaining thermodynamics parameters; namely the standard Gibbs free energy change (*ΔG°*) (kJ/mol), the standard enthalpy change (*ΔH°*) (kJ/mol), and standard entropy change (*ΔS°*) (kJ/mol/K)^42^ included in Eqs. (10) and (11), at varying temperatures, from 25 °C to 45 °C (298 to 318 K).

12 $$\:Ln\:\frac{{q}_{e}}{{C}_{e}}=\:\frac{\varDelta\:S^\circ\:}{R}-\:\frac{\varDelta\:H^\circ\:}{RT},$$

13 $$\Delta G^\circ {\text{ }} = {\text{ }}\Delta H^\circ {\text{ }} - {\text{ }}T{\text{ }}\Delta S^\circ$$

where *C*_*e*_ is the equilibrium concentration (mg/L), *q*_*e*_ is the amount of phosphorus adsorbed (mg/g), T is the temperature (K), and R is the universal gas constant (8.314 J/mol·K).

### Adsorbent regeneration

In order to investigate the reusability potential of MF and MFBCs, two successive cycles of adsorption were performed. The adsorption process was carried out at the optimum conditions obtained from batch experiments, then MF and MFBCs particles were separated using a magnet. The collected particles were washed by distilled water to remove the adsorbed phosphorus. After that, the washed particles were used for a second adsorption cycle and the removal efficiency of phosphorus is evaluated at the same optimum conditions.

## Results and Discussion

### Results of batch experiments

#### Effect of pH

**Fig. 1:**
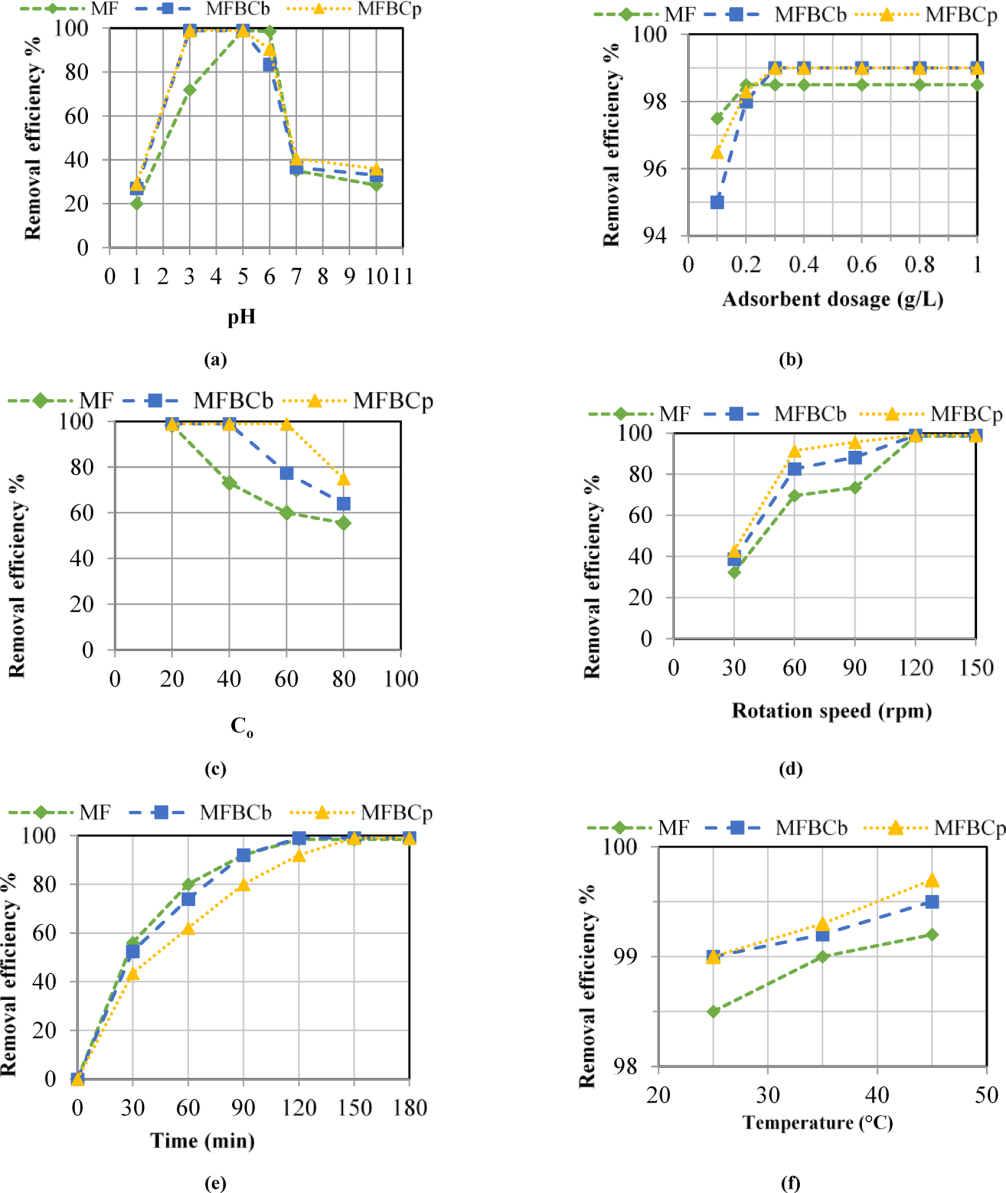
Effect of: (**a**) pH, (**b**) adsorbent dose, (**c**) initial phosphorus concentration, (**d**) rotation speed, (**e**) retention time, and (**f**) temperature on phosphorous removal.

The charge properties and strength of the adsorbents can be affected by the pH of the solution. In this batch, initial adsorbent dose of MF of 1 g/L, initial phosphorus concentration of 20 mg/L, retention time of 150 min, rotation speed of 120 rpm, and temperature of 25 °C were used. As shown in Fig. 1(a), it was noticed that as the pH value increased from 1 to 5, the removal efficiency increased and reached 99% and decreased slightly at pH 6 with 98.5%. When the pH value exceeded 6, the removal efficiency decreased significantly to 35% at pH 7, and continued to decreased but at a slight rate until it reached its minimum value of 28.5% at pH 10. When MFBCs were applied with initial adsorbent dose of 1 g/L and the same initial conditions for the other parameters, the removal efficiency reached 99% at pH 3 and remained constant until pH 5. After that, the removal efficiency decreased to 83.5% and 90.5% at pH 6 for MFBC_b_ and MFBC_p_, respectively, and then it continued to decrease but at a slight rate similar to the MF. This depict that when the pH is below 7, the surface charge of the adsorbent particles becomes positive, so that more phosphorus particles is adsorbed due to the increase in electrostatic attraction with the anionic phosphorus molecules. The optimum values of pH are 5 and 3 for MF and MFBCs, respectively. For field applications, it is recommended to apply the higher pH value to minimize chemical usage while attaining acceptable removal efficiency.

#### Effect of adsorbent dose

In this batch, optimum value of pH 5, initial phosphorus concentration of 20 mg/L, retention time of 150 min, rotation speed of 120 rpm, temperature of 25 °C, and adsorbent dose of MF varying between 0.2 and 1 g/L were used. As shown in Fig. 1(b), as the adsorbent dose of MF was increased from 0.1 to 0.2 g/L, the removal efficiency increased from 97.5 to 98.5% and remained constant for higher adsorbent doses tested. When applying MFBCs at pH 3 and the same initial conditions used for MF, the removal efficiency reached 99% at adsorbents’ dose of 0.3 g/L, then the removal efficiency remained constant when higher adsorbents’ doses were applied. The effect of changing the adsorbent dose on the efficiency of phosphorus removal can be explained by the fact that the lower the concentration of the adsorbent, the faster its pores are saturated, and thus low removal efficiencies are achieved. Accordingly, when the concentration of the adsorbent dose was increased, larger quantities of phosphorus were adsorbed, and thus an increase in the removal efficiency was obtained. However, a maximum removal efficiency is achieved once all phosphorous particles in the solution are adsorbed and increasing the adsorbent dose is no longer required. The optimum values of the adsorbent dose are 0.2 g/L for MF and 0.3 g/L for MFBCs.

#### Effect of initial phosphorus concentration

This batch was performed to investigate the effect of changing the initial phosphorous concentrations, ranged from 20 to 80 mg/L, on phosphorous removal efficiency at the optimum values of pH and adsorbent dose obtained from the previous batches, retention time up to 180 min, rotation speed of 120 rpm, and temperature of 25 °C. As shown in Fig. 1(c), it was noticed that the maximum removal efficiencies were 98.5% and 99% for MF and MFBCs, respectively, at initial concentration of 20 mg/L for all adsorbents. Thereafter, at initial concentration of 40 mg/L, the percentage of phosphorus removal decreased to 73% for MF and continued to decrease for higher initial phosphorus concentrations. On the other hand, the removal efficiency using MFBCs remained the same. Following, at initial concentration of 60 mg/L, the percentage of phosphorus removal decreased to 77.5% for MFBC_b_ and continued to decrease for higher initial phosphorus concentrations. However, the removal efficiency using MFBC_p_ remained the same. When increasing the initial concentration to 80 mg/L, the removal efficiency using MFBC_p_ decreased to 75%. This is due to the ratio of the vacant active sites of the adsorbent to the adsorbate molecules affects the removal efficiency of the phosphorus adsorption process. At a constant adsorbent dose, the vacant active adsorption sites are limited and by increasing the initial phosphorous concentration, the active sites became saturated and, hence, the removal efficiency decreased. The optimum initial phosphorous concentration values are 20, 40, and 60 mg/L for MF, MFBC_b_, and MFBC_p_, respectively.

#### Effect of rotation speed

This batch was performed at different rotation speeds; 30, 60, 90, 120, and 150 rpm; a retention time of 120 min, and temperature of 25 °C with the optimum values of pH, adsorbent dose, and initial phosphorus concentration obtained from previous batches. As shown in Fig. 1(d), for both MF and MFBCs, the removal efficiency was about 40% for all adsorbents at rotation speed of 30 rpm. As the rotation speed increased to 90 rpm, the removal efficiency improved to more than 60% for all adsorbents and reached a maximum of 98.5% and 99% for MF and MFBCs, respectively, at 120 rpm. By increasing the rotation speed to 150 rpm, no further improvement was obtained. The optimum rotation speed value of 120 rpm for both MF and MFBCs is applied for the following batch.

#### Effect of retention time

This batch was performed at different retention times 30, 60, 90, 120,150 and 180 min with the optimum values of pH, adsorbent dose and initial phosphorus concentration obtained from the previous batches, rotation speed of 120 rpm, and temperature of 25 °C. As shown in Fig. 1(e), it was noticed that as the time increased from 30 to 120 min, the adsorption process progressed and the removal efficiency increased until it reached 98.5% and 99% of MF and MFBC_b_, respectively. While for MFBC_p_, as the time increased from 30 to 150 min, the adsorption process progressed and the removal efficiency increased until it reached 99%. By the end of testing period, the removal efficiency remains constant for all cases. The removal rate was rapid in the first 30 min because at the beginning the active sites on the surface of the adsorbent particles were vacant. Over time, the adsorbent surface becomes saturated with phosphorus and, hence, the removal rate decreased. The optimum retention time value is 120 min for both MF and MFBC_b_, while the optimum retention time value is 150 min for MFBC_p_.

#### Effect of temperature

This batch was performed at different temperature values; 25, 35, and 45 °C; with the optimum values of pH, adsorbent dose, initial phosphorus concentration, retention time, and rotation speed obtained from previous batches. As shown in Fig. 1(f), for both MF and MFBCs, it was noticed that at temperature of 25 °C, removal efficiency of 98.5% and 99% for MF and MFBCs, respectively. By increasing the temperature, the removal efficiency was increased gradually until it reached 99.2%, 99.5%, and 99.7% for MF, MFBC_b_, and MFBC_p_, respectively, at a temperature of 45 °C. This indicates that the adsorption process is an endothermic reaction. Accordingly, the optimum value temperature shall be 45 °C for all adsorbents. However, it is recommended to adopt the 25 °C for field applications since the improvement in phosphorus removal efficiency is incomparable to heating costs required to provide the 45 °C.

### Adsorption kinetics

**Fig. 2:**
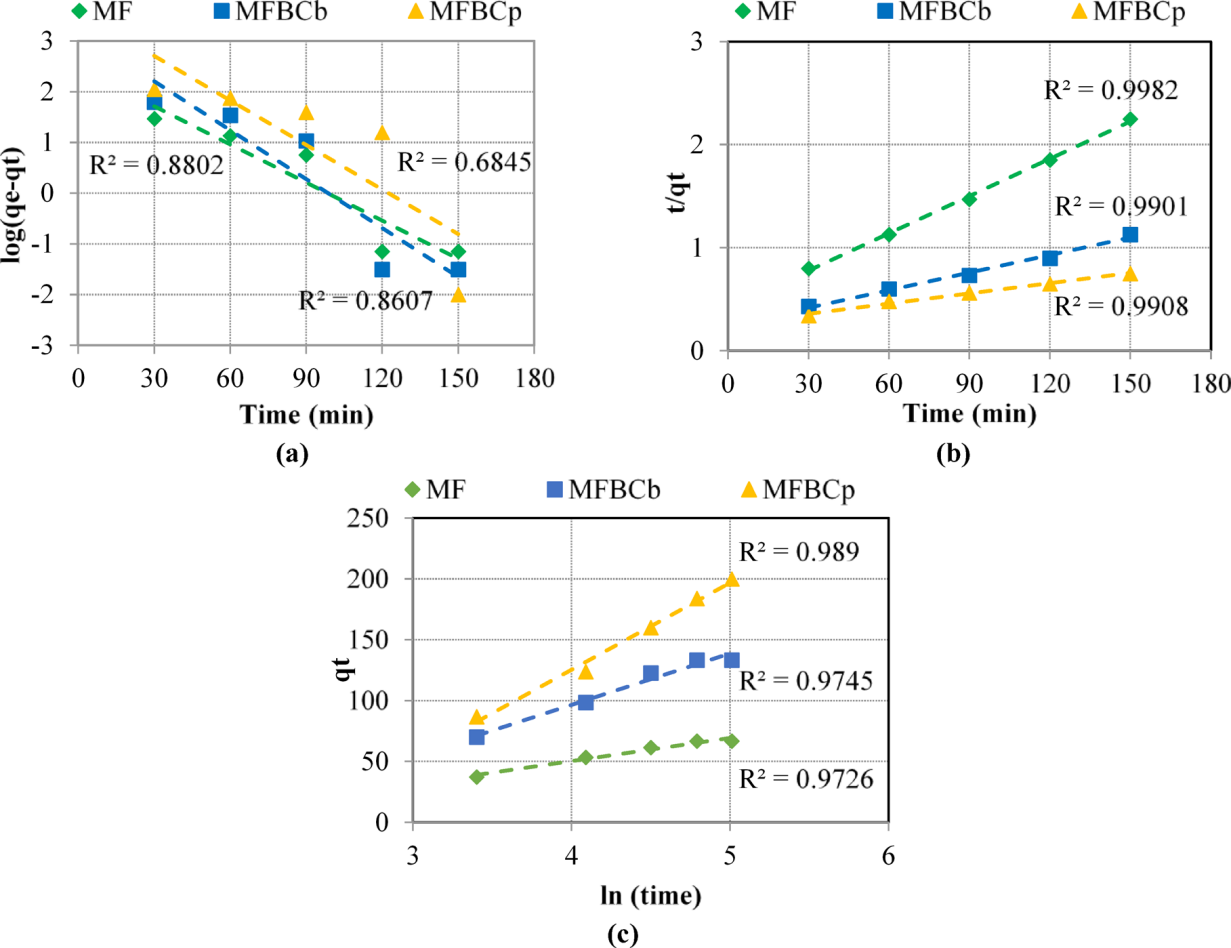
Adsorption kinetics of phosphorous onto all adsorbents (**a**) PFO, (**b**) PSO, and (**c**) Elovich equation.

The adsorption kinetics, which indicate the uptake rate of phosphorus ions from the water, were studied using three models; namely pseudo-first order (PFO), pseudo-second order (PSO), and Elovich equation to fit the adsorption data. The kinetic parameters and correlation coefficients (R^2^) for each adsorbent were determined as shown in Fig. 2(a-c) and summarized in Table 5. The calculated equilibrium phosphorus adsorption capacities on MF and MFBCs were close to the experimental data. The PSO model provided the highest R^2^ values with 0.9982, 0.9901, and 0.9908 for MF, MFBC_b_, MFBC_b_, respectively. These results suggest that chemisorption plays a significant role in this adsorption process.

**Table 5:** Kinetic parameters.

Adsorption kinetics	Kinetic parameters		MF	MFBC_b_	MFBC_*p*_
PFO model	k_1_ Min^− 1^		25.1*10^− 3^	32.1*10^− 3^	29.3*10^− 3^
	q_e, cal_ mg/g		11.775	23.712	35.874
	R^2^		0.8802	0.8607	0.6845
PSO model	K_2_ g/mg.min		3.536*10^− 4^	1.31*10^− 4^	4.205*10^− 5^
	q_e, cal_ mg/g		82.645	175.439	303.03
	R^2^		0.9982	0.9901	0.9908
Elovich equation	α mg/g.min		4.788	7.352	7.531
	β g/mg		0.053	0.024	0.014
	R^2^		0.9726	0.9745	0.989

### Adsorption isotherms and adsorption capacity

**Fig. 3:**
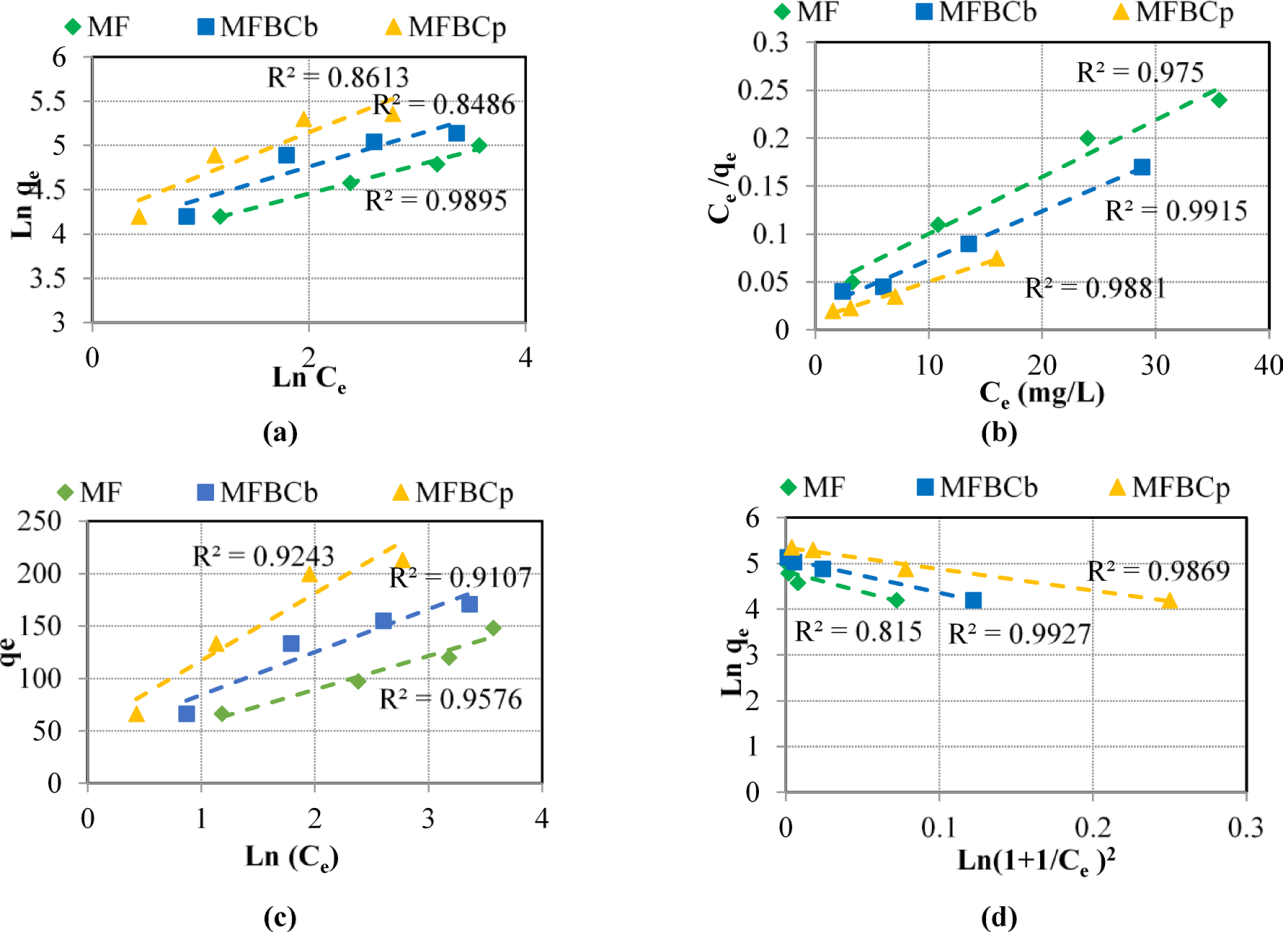
Adsorption isotherms of phosphorous onto all adsorbents (**a**) Freundlich, (**b**) Langmuir, (**c**) Temkin, and (**d**) Dubinin-Radushkevich (D-R).

The adsorption isotherms are used to describe the solid-liquid adsorption system. The adsorption isotherm is considered a basic indicator for choosing the type of adsorbent suitable for the absorption of a particular pollutant. It also contributes in determining the adsorption capacity. Figures 3(a-d) represent the experimental results using the linearized form of Freundlich, Langmuir, Temkin, and D-R isotherm equations, respectively. Each isotherm equation has specific adsorption assumptions. Adsorption occurs on a heterogeneous surface according to Freundlich assumptions, while Langmuir expresses the maximum limiting adsorption at a given number of accessible sites on the adsorbent surface, with the same energy available at all adsorption sites. Temkin assumes that the adsorption heat decreases linearly as the surface of the adsorbent is covered. D-R isotherm represents the influence of the surface porous of the adsorbent^10^. The correlation coefficients (R^2^) were determined for each adsorbent as shown in Fig. 3 and the results of equilibrium studies on phosphorus removal using MF and MFBCs are summarized in Table 6.

The Freundlich isotherm better described the adsorption behavior of MF at R^2^ of 0.9895, which indicates that the process of phosphorous adsorption is a multilayer adsorption process on heterogeneous sites^37^. The high *K*_*F*_ value, 44.7 mg/g, and 1/*n* value < 1 (0.32) show high adsorption capacity and favorable adsorption capabilities with strong binding at low concentrations. On the other hand, the adsorption behavior of MFBC_b_ was best described by the Dubinin-Radushkevich (D-R) isotherm at R^2^ of 0.9927, which suggests that the porous structure of the adsorbent surface affects the adsorption process^10^. The *β* value can be used to calculate the mean adsorption energy (E), Eq. (14), which refers to the mean free energy of adsorption per mole of adsorbate as it moves to the surface.

14 $$\:E=\frac{1}{\sqrt{2\beta\:}}.$$

If the E value is less than 8 kJ/mol, the adsorption process is physisorption, while E value greater than 16 kJ/mol indicates a chemisorption adsorption system^23^. E value between 8 − 16 kJ/mol may suggest ion exchange. The E value of phosphorus adsorption on MFBC_b_ is 0.26 kJ/mol (< 8 kJ/mol) indicating adsorption is mainly via physisorption.

The adsorption behavior of MFBC_p_ was best fitted by the Langmuir isotherm at R^2^ of 0.9881 which indicates that the process of phosphorous adsorption happens as s monolayer form where the surface of adsorbent is energetically homogeneous^38^.

The high *K*_*L*_ factor of the Langmuir model (0.34 L/mg) suggests strong adsorbate-adsorbent interaction. Also, it can be used to determine the dimensionless separation factor (*R*_*L*_), Eq. (15), to evaluate whether adsorption is favorable.

15 $$\:{R}_{L}=\frac{1}{1+{K}_{L}{C}_{0}},$$

where *C*_*0*_​ is the initial concentration of adsorbate (mg/L). If the *R*_*L*_ value equals 1 then the adsorption process is linear, while zero *R*_*L*_ value refers to irreversible adsorption. *R*_*L*_ value between 0 − 1 indicates favorable adsorption^43^. The *R*_*L*_ value of phosphorus adsorption on MFBC_p_ is 0.01 demonstrating favorable adsorption process.

The maximum corresponding adsorption capacities obtained from this study are 44.70 mg/g, 164.137 mg/g, and 256.41 mg/g for MF, MFBC_b_, MFBC_p_, respectively, compared to other magnetic and non-magnetic adsorbents. This superior performance is attributed to the synergy between MnFe₂O₄ nanoparticles and activated carbon, which provides a high surface area and abundant active sites. Beside the strong electrostatic interaction and ligand exchange mechanism facilitated by the Fe-O and Mn-O bonds. Adsorption capacity of different magnetic and non-magnetic nanoparticles from previous studies compared to the adsorbents considered in this research are presented in Table 7.

According to Table 7, MFBCs, especially MFBC_p_, looks promising regarding phosphorus removal, whilst MF shows moderately average adsorption capacity compared to other magnetic nanoparticles. Additionally, Magnetic nanoparticles (e.g., MnFe₂O₄@AC) exhibit higher adsorption capacities compared to non-magnetic adsorbents such as biochar and activated carbon. This is attributed to their high surface area, enhanced porosity, and the presence of active sites such as Fe-O and Mn-O bonds that enable strong interaction with phosphate ions. Also, magnetic nanoparticles can be easily separated from aqueous solutions using an external magnetic field, reducing post-treatment costs and enhancing their practicality in real-world applications. While magnetic nanoparticles are reusable, one of negative points are their adsorption efficiency may decline after multiple cycles due to partial saturation of active sites or structural degradation. Furthermore, as seen from Table 7, Non-magnetic adsorbents typically exhibit lower adsorption capacities compared to magnetic nanoparticles due to their limited active sites and weaker interactions with phosphate ions, whish is negative point. Also, Non-magnetic adsorbents require additional filtration or centrifugation steps for recovery, increasing operational complexity and cost.

**Table 6:** Isotherm parameters.

Adsorption isotherms	Parameters		MF	MFBC_b_	MFBC_*p*_
Freundlich	*K*_*F*_ mg/g		44.70	54.60	64.72
	*n* -		3.11	2.74	2.04
	*R* ^*2*^ -		0.9895	0.8486	0.8613
Langmuir	*q*_*m*_ mg/g		169.49	196.08	256.41
	*K*_*L*_ *L/mg*		0.14	0.24	0.34
	*R* ^*2*^ -		0.975	0.9915	0.9881
Temkin	*b* *J/mol*		77.49	60.65	38.64
	*K*_*T*_ L/g		2.23	2.89	2.27
	*R* ^*2*^ -		0.9576	0.9107	0.9243
Dubinin-Radushkevich (D-R)	*q*_*d*_ mg/g		124.786	164.137	210.04
	*β* mol^2^/J^2^		8.948	7.439	4.691
	*R* ^*2*^ -		0.815	0.9927	0.9869

**Table 7:** Comparison of the adsorption capacities of phosphorous using various magnetic and non-magnetic nanoparticles from previous studies.

Materials	Type	Adsorption capacities (mg/g)	References
Peanut shell BC	Non-magnetic	7.57	^44^
Different Biochars	Non-magnetic	8.8–13.9	^45^
non-magnetic (WT) montmorillonite	Non-magnetic	10.4-14.99	^46^
Pine BC	Non-magnetic	14.49	^44^
Activated Carbon (AC)	Non-magnetic	18.45	^47^
Crawfish BC	Non-magnetic	70.9	^44^
Soybean stover BC	Non-magnetic	90.9	^44^
Magnesium-modified ceramsite from iron tailings	Magnetic	10.24	^48^
and magnetically modified (WMT) montmorillonite	Magnetic	10.36–13.21	^46^
MgFe_2_O_4_-biochar based lanthanum alginate beads	Magnetic	26.83	^27^
NiFe_2_O_4_	Magnetic	39.3	^26^
Fe_3_O_4_@chitosan core-shell	Magnetic	48.2	^29^
Nano zero valent iron	Magnetic	53.76	^10^
ZnFe_2_O_4_@activated carbon composite	Magnetic	91.8	^25^
La(OH)_3_/CoFe_2_O_4_	Magnetic	104.01	^28^
La_2_O_2_CO_3_/Fe_2_O_3_ composite	Magnetic	134.82	^31^
MF	Magnetic	44.7	This work
MFBC_b_	Magnetic	164.137	This work
MFBC_p_	Magnetic	256.41	This work

### Adsorption mechanism

The adsorption of phosphorus onto the magnetic nanoparticles (MF) and biochar composites (MFBC_b_ and MFBC_p_) involves multiple stages, including external diffusion, surface adsorption, and intra-particle diffusion. These stages were analyzed using the intra-particle diffusion model and Boyd kinetic model to better understand the controlling steps of the adsorption process. The intra-particle diffusion model indicates that the adsorption process occurs in three distinct stages; Stage 1: Film diffusion, where phosphorus ions are transported from the bulk solution to the external surface of the adsorbents. Stage 2: Intra-particle diffusion, where phosphorus ions diffuse into the pores of the adsorbents. Stage 3: Adsorption equilibrium, where phosphorus ions are adsorbed onto the active sites in the interior of the adsorbent^49–51^.

**Fig. 4:**
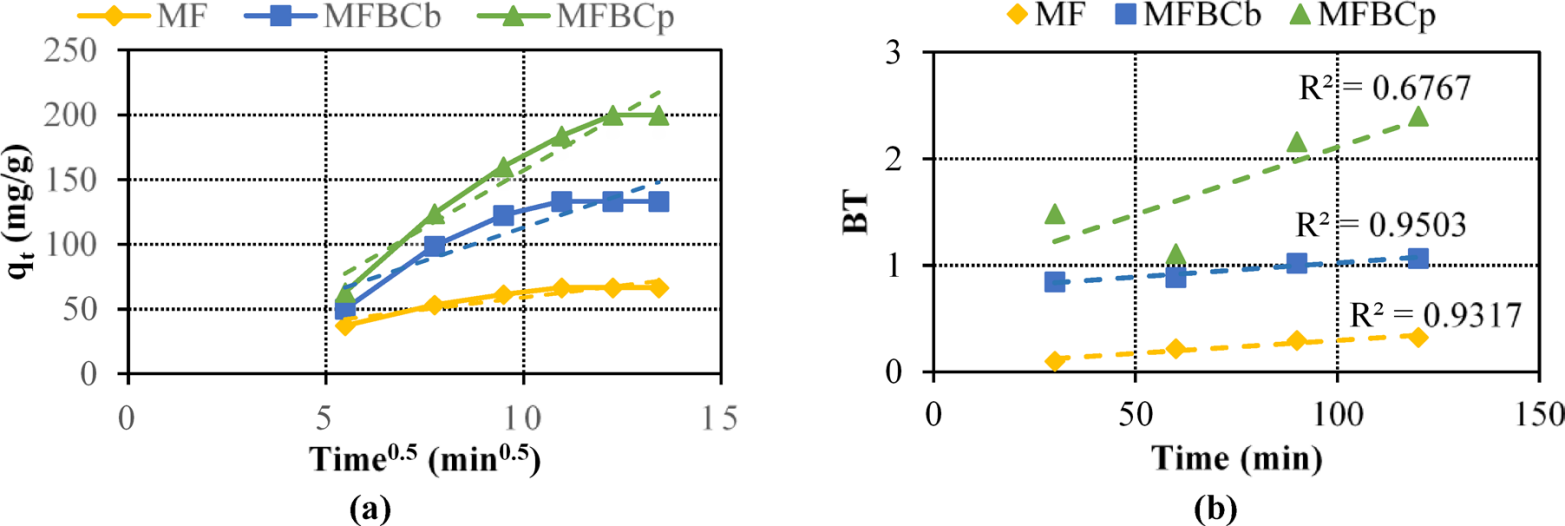
Adsorption mechanisms of phosphorous onto all adsorbents: (**a**) intra-particle diffusion model and (**b**) Boyd kinetic model.

The plots for intra-particle diffusion showed multilinear behavior, confirming the presence of multiple adsorption stages^52^. However, the linear portions did not pass through the origin, suggesting that intra-particle diffusion is not the sole rate-limiting step. Figure 4(a) displays the plots of the intra-particle diffusion model produced for phosphorus adsorption on MF and MFBCs particles. The x-axis represents time ^0.5^ and the y-axis represents the phosphorus adsorption capacity. Three linear sections can be seen in the plots, indicating that the adsorption process happened in three steps. Diffusion effects from films or surfaces are typically responsible for the initial steeper Sect.^53^. The progressive adsorption stage, where intra-particle or pore diffusion is rate-limiting, is described in the second linear section. The last adsorption equilibrium stage, for which the intra-particle diffusion began to slow down as a result of attaining the equilibrium, is responsible for the third section.

The Boyd kinetic model was applied to distinguish between film diffusion and intra-particle diffusion. The linear plots of the Boyd model, which did not pass through the origin, confirm that surface diffusion is the primary rate-limiting step in the adsorption of phosphorus onto the adsorbents^54^. According to Fig. 4(b); the Boyd kinetic model plots are linear but do not pass through the origin for all studied adsorbents, MF and MFBCs, which suggests that the surface diffusion is the main governor of phosphorus adsorption on MF and MFBCs particles.

The adsorption of phosphorus is primarily governed by chemisorption, as evidenced by the pseudo-second-order kinetic model’s excellent fit (R² > 0.99 for all adsorbents). This indicates that phosphorus ions interact with functional groups, such as hydroxyl and carboxyl groups, present on the surface of the adsorbents. Additionally, Electrostatic interactions between the negatively charged phosphate ions (PO₄³⁻) and the positively charged active sites on the adsorbents play a significant role, particularly at lower pH values. The formation of inner-sphere complexes through ligand exchange further enhances adsorption efficiency^55^.

### Thermodynamics studies

**Fig. 5:**
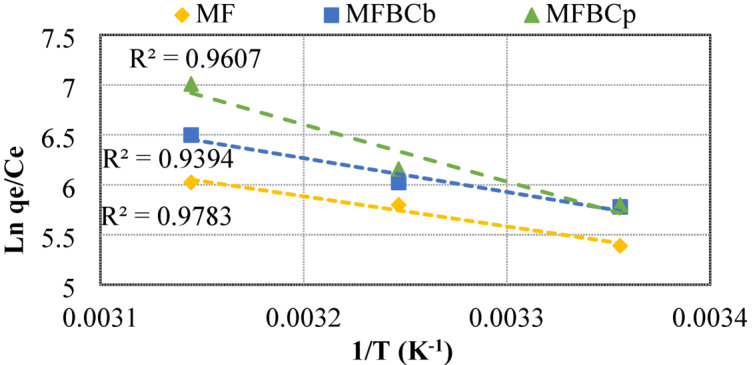
Van’t Hoff chart of the adsorption of phosphorus.

Thermodynamics describes the behavior of matter and the transformation between different forms of energy. The data obtained from experiments of phosphorus adsorption at temperatures ranging from 25 °C to 45 °C (298 to 318 K), used to determine parameters of thermodynamics. As shown in Fig. 5, by plotting Ln *(q*_*e*_ */ C*_*e*_) versus 1/T, the values of enthalpy (*ΔH°*) were calculated from the slope and the values of entropy (*ΔS°*) were calculated from the interception of a Van’t Hoff chart. Table 8 summarizes the values of thermodynamic parameters ΔG°, ΔH°, and ΔS°. The positive enthalpy values indicate that phosphorus adsorption by MF and MFBCs is an endothermic process. The positive entropy (*ΔH****°***) values reflect excess randomness at the solid-solution interface through the adsorption process. The negative values of Gibbs free energy (*ΔG****°***) decreased as the temperature increased which indicate that the process was spontaneous and as temperatures increased, the spontaneity of the adsorption process increased.

**Table 8:** Thermodynamics parameters.

T (°C)	T (K)	Adsorbent	ΔG° (kJ/mol)	ΔH° (kJ/mol)	ΔS° (kJ/mol/K)
25	298	MF	-38.476	25.085	129.2
35	308		-39.769	25.085	129.2
45	318		-41.061	25.085	129.2
25	298	MFBC_b_	-42.368	28.177	142.27
35	308		-43.791	28.177	142.27
45	318		-45.213	28.177	142.27
25	298	MFBC_p_	-61.587	47.479	206.83
35	308		-63.656	47.479	206.83
45	318		-65.724	47.479	206.83

### Characterization of MF and MFBCs

#### Scanning electron microscopy (SEM) analysis

**Fig. 6:**
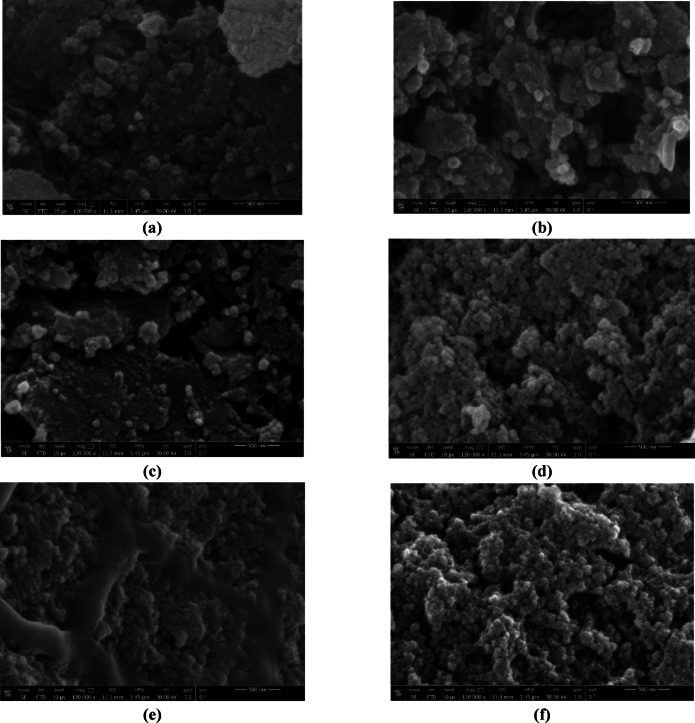
SEM characterization about particles formed on: (**a**) MF, (**c**) MFBCb, and (**e**) MFBC_b_ before reaction, and (**b**) MF, (**d**) MFBCb, and (**f**) MFBC_b_ after reaction with phosphorus.

The morphology of the adsorbents’ surface was examined using the SEM test, where SEM images observed changes in the morphology of the adsorbents before (unloaded) and after (loaded) the adsorption process, see Figs. 6(a) − 6(f). The SEM image of unloaded MF, Fig. 6(a), shows solid and smooth surface with few porous holes. On contrary, the SEM images of unloaded MFBC_b_, MFBC_p_, Figs. 6(c) and 6(e), respectively, show that both MFBCs have irregular, granular, and tough surfaces with porous holes. This may explain the low removal efficiency of phosphorus using MF compared to MFBCs. After the adsorption process, Figs. 6(b), 6(d), and 6(f) of loaded MF and MFBCs show that the porous holes on adsorbents’ surfaces were filled. The comparison of the SEM images of MF, and MFBCs before and after phosphorus uptake at different magnification scales reveal significant changes in the morphology and proves that the adsorption process has occurred.

#### Energy-dispersive X-ray spectroscopy (EDX) analysis

**Fig. 7:**
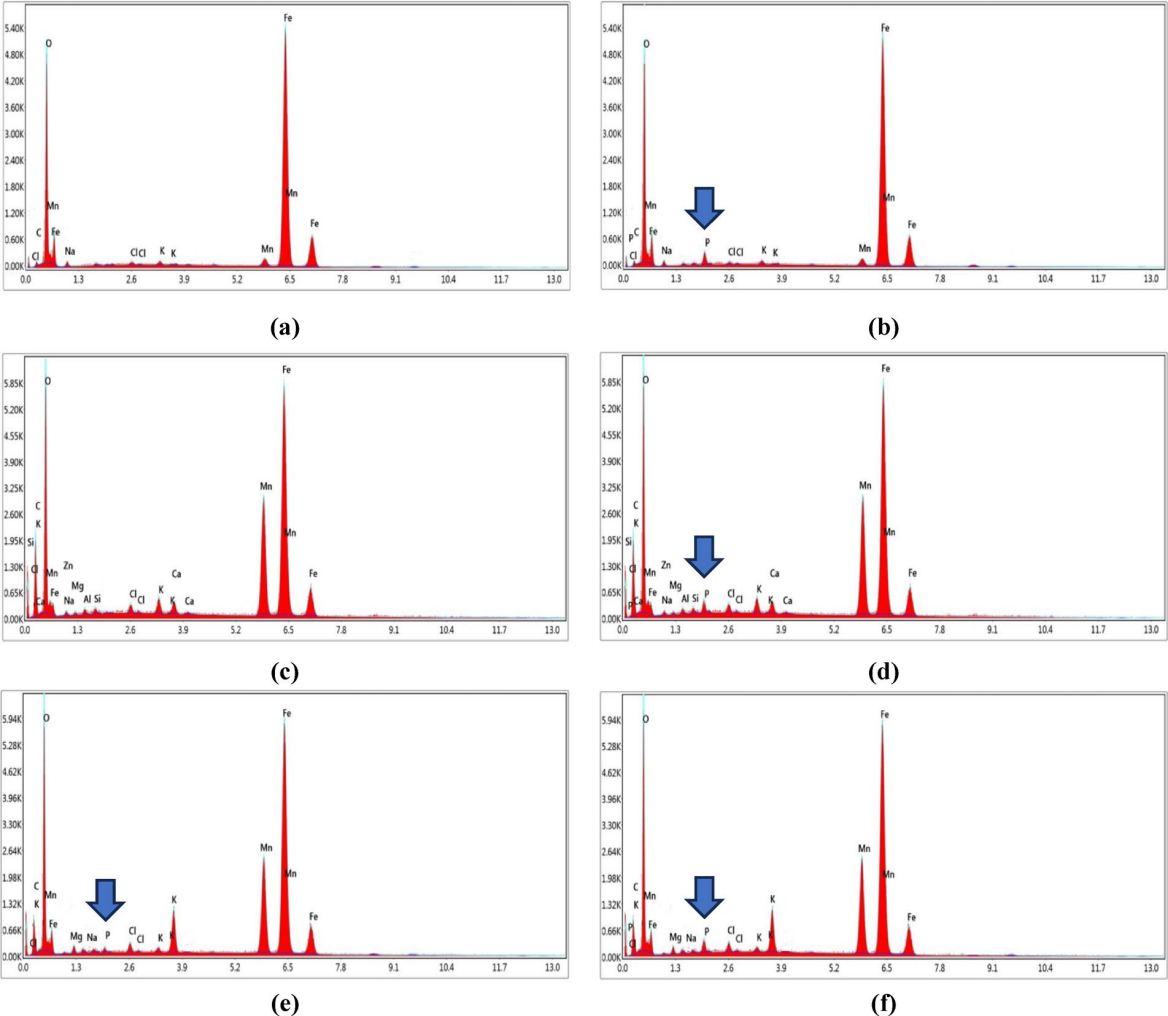
EDX analysis results: (**a**) MF, (**c**) MFBCb, and (**e**) MFBC_b_ before reaction, and (**b**) MF, (**d**) MFBCb, and (**f**) MFBC_b_ after reaction with phosphorus.

EDX analysis was used to determine the elemental composition and chemical characterization of adsorbents’ samples before and after the adsorption process. Figures 7(a)–(f) show the EDX spectra for MF, and MFBCs, before (unloaded) and after (loaded) the adsorption process. Figures 7(a), (c), and (e) of unloaded adsorbents proved no index for presence of any phosphorus ions before the adsorption process. After the adsorption process, the EDX analysis proved phosphorus uptake on MF, and MFBCs. Figures 7(b), (d), and (f) revealed the accumulation of phosphorus onto the surface of MF, and MFBCs particles. Moreover, the amount of phosphorous measured on loaded MFBC_p_ is greater than that measured for loaded MFBC_b_, while loaded MF has the least amount of phosphorous. These results support the adsorption capacity results obtained in the adsorption isotherms.

#### Fourier-transform infrared spectroscopy (FTIR) analysis

**Fig. 8:**
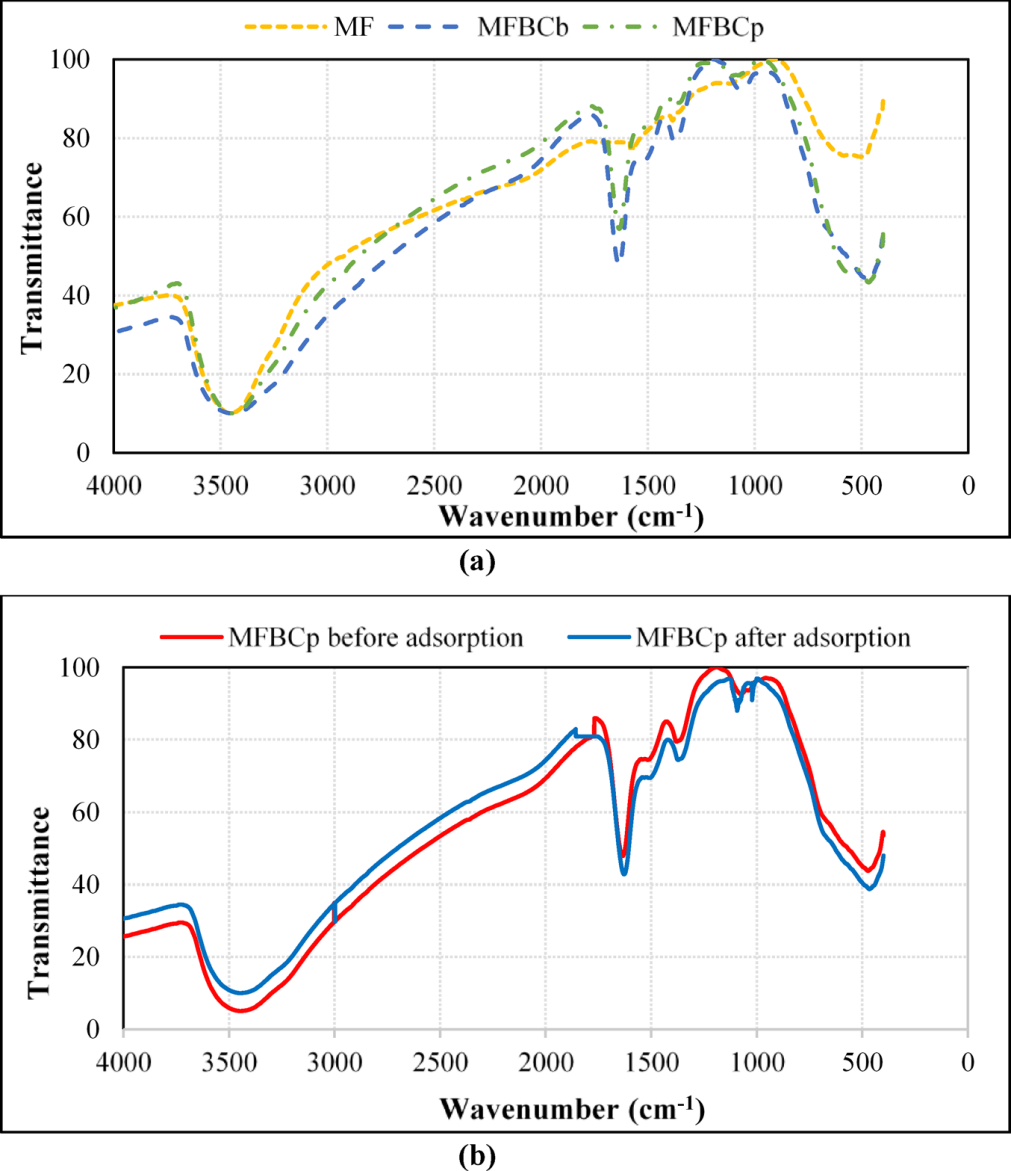
(**a**) FTIR analysis results for MF and MFBCs before adsorption. (**b**) FTIR spectra of the MFBCp before and after phosphate adsorption.

Fourier transform infrared (FTIR) spectroscopy was used to characterize and analyze the chemical structure of the samples and the surface functional groups of the composites by the KBr pellet technique. The spectra were collected between 400 and 4000 cm^− 1^. The corresponding FTIR spectra of MF and MFBCs shown in Fig. 8 were used to identify the functional groups on the external surface of the unloaded MF and MFBCs and confirm the successful formation of MF and MFBCs hybrids.

Overall, all spectrums showed a broad band in the range 3443–3453 cm^− 1^, which may be associated with the stretching and bending vibration of the O-H groups of crystal and adsorbed water which is associated with the Fe hydroxyl on the Fe oxides surface^56,57^. The band around 1644 cm^− 1^ in the pattern of MFBCs were attributed to the aromatic C = C and C = O stretching vibrations. The lignin structure of peanuts and bagasse branches may be responsible for these organic functional groups^58^. The bands appeared at 1375–1380 cm^− 1^ characterized to the binding vibrations of C-C and C-O of the aromatic rings^59^. Bands at approximately 451 and 595 cm^− 1^ may be identified as the IR absorption bands of MF corresponded to the formation of Fe-O and Mn-O bonds, confirming the successful synthesis of MF and the successful incorporation of Mn/Fe oxide nanoparticles in the external surface of the modified BC materials^60^. For MnFe_2_O_4_, the vibrations of the octahedral group are mostly responsible for the IR bands between 600 and 400 cm^− 1^^61^. Comparative analysis of the FTIR spectra before and after phosphate adsorption by MFBCp, Fig. 8(b), shows new peaks appeared in the region 1050–1100 cm⁻¹, corresponding to P–O stretching vibrations, confirming the presence of adsorbed phosphate ions^57^. Furthermore, the Fe-O and Mn-O bands (451 cm⁻¹ and 595 cm⁻¹) showed slight shifts and intensity changes, suggesting that phosphorus adsorption influenced the metal-oxygen bonds in manganese ferrite^57,62^. After adsorption, the signals at 481.89 cm^− 1^ enhanced, which may be due to the addition of P-O. Also, a significant reduction in the intensity of the O-H stretching band (3443–3453 cm⁻¹) was observed, indicating interaction between phosphorus species and hydroxyl groups^63^. These findings provide clear evidence of the adsorption of phosphorus on the surface of the magnetic nanoparticles and biochar composites.

### Regeneration study

**Fig. 9:**
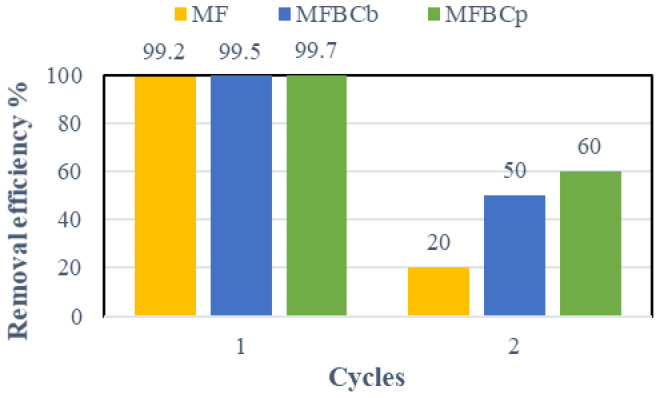
Results of regeneration study.

Regeneration is a desirable trait for an effective adsorption. The reusability potential of MF and MFBCs was evaluated by restoring the filtered adsorbents after the adsorption process and applying them for a second adsorption cycle at the same optimum conditions obtained from the batch experiments. As shown in Fig. 9, the removal efficiency of all adsorbents was reduced dramatically for the second cycle.

The removal efficiency using MFBC_p_ decreased from 99.7 to 60%, while using MFBC_b_ showed a reduction from 99.5 to 50%, and lastly comes the MF with a reduction from 99.2 to 20%. This may be attributed to the fact that some adsorbates remained on the adsorbents’ surfaces causing fewer active sites to be available for adsorbing phosphorus during the second cycle and, hence, the adsorption capacity of regenerated adsorbent particles is reduced. In addition, the MFBC_p_ continued to show higher adsorption capacity than that of the MFBC_b_. Accordingly, it is advised to increase the adsorbent dose for the second cycles using MFBCs by adding an adjusted amount of fresh MFBCs to obtain better removal efficiency of phosphorous. However, for MF seems to be of less attraction to be regenerated for a second adsorption cycle.

There are reasons for low removal rate during the second cycle, during the first regeneration cycle, not all adsorbed phosphorus is effectively desorbed from the active sites of the adsorbent. Residual phosphorus may block adsorption sites, reducing the adsorption capacity in subsequent cycles^64^. Additionally, magnetic nanoparticles (e.g., MnFe₂O₄@AC) may undergo structural changes or degradation during the regeneration process, particularly under harsh chemical or thermal conditions. This can reduce the availability of active sites for phosphorus adsorption^65,66^. Likewise, Prolonged exposure to alkaline or acidic desorption agents may lead to the leaching of Fe or Mn ions, reducing the magnetic properties and adsorption efficiency of the nanoparticles^67,68^. Else, Organic or inorganic impurities present in the water may accumulate on the adsorbent surface, blocking active sites and interfering with the adsorption of phosphorus during subsequent cycles^69^.

To address the limitations of current regeneration methods, some approaches are suggested for further investigation like A combination of weak acidic and alkaline solutions can be used sequentially to improve desorption efficiency^70^. Or, using chelating agents (e.g., EDTA or citric acid) can enhance the desorption of phosphorus by breaking strong bonds between phosphorus and the adsorbent surface^71^. Too, Bai et al., reported thermal regeneration at moderate temperatures (e.g., 300–500 °C) can remove residual phosphorus without causing significant structural damage to the adsorbent. This method may also restore the magnetic properties of nanoparticles^70^. Finally, for future studies, investigating alternative magnetic adsorbents with stability and reusability, such as graphene-based magnetic composites or doped ferrites, could provide better performance in repeated cycles.

The final disposal of phosphorus-adsorbed magnetic nanoparticles (MnFe₂O₄@AC) is a significant aspect of ensuring environmental safety and sustainability. Phosphorus-adsorbed nanoparticles can be regenerated using desorption agents such as alkaline solutions (e.g., NaOH or KOH) to release adsorbed phosphorus. The desorbed phosphorus can then be recovered and reused in agriculture as phosphate fertilizers^72^. Furthermore, phosphorus-adsorbed nanoparticles can be directly applied to agricultural soils as a slow-release fertilizer. The magnetic nanoparticles, when incorporated into soil, gradually release phosphorus, enhancing nutrient availability for crops without causing nutrient leaching into water bodies. The biochar component of MnFe₂O₄@AC can also improve soil health by enhancing water retention, microbial activity, and carbon sequestration^73,74^. Additionally, spent nanoparticles can be immobilized using cement or other binding agents to prevent leaching of phosphorus or metal ions into the environment. This method is particularly useful for disposing of nanoparticles in landfills or construction applications^75^. Also, Incineration of phosphorus-loaded nanoparticles can convert phosphorus into phosphate ash, which can be recovered and used as raw material for phosphate fertilizers^76^. The integration of phosphorus-adsorbed nanoparticles into a circular economy has been emphasized. By recovering phosphorus for reuse and regenerating the nanoparticles for multiple adsorption cycles, waste can be minimized, and sustainability can be maximized.

## Conclusions

This study demonstrated the potential of manganese ferrite (MF) and its biochar-supported composites (MFBCb and MFBCp), derived from agricultural wastes, as efficient adsorbents for phosphorus removal from aqueous solutions. MFBCp exhibited the highest adsorption capacity (256.41 mg/g), followed by MFBCb (164.14 mg/g) and MF (44.7 mg/g), confirming the enhanced performance of biochar-supported composites. The adsorption process was best described by the pseudo-second-order kinetic model, suggesting chemisorption, while isotherm studies highlighted monolayer adsorption for MFBCb and MFBCp. Mechanistic and thermodynamic analyses identified surface diffusion as the rate-limiting step and confirmed that the process is endothermic and spontaneous. Regeneration studies revealed that MFBCb and MFBCp maintained reasonable efficiency in a second cycle (50% and 60%, respectively), while MF showed poor reusability. These findings highlight the suitability of MFBCp for practical wastewater treatment applications, combining high adsorption performance with sustainable use of agricultural byproducts. Future work should focus on improving regeneration efficiency and testing these adsorbents under real-world conditions.

## Data Availability

All data generated or analyzed during this study are included in this published article.

## Abbreviations

°C: Celsius
BC: Biochar
BC_b_: Biochar from bagasse
BC_p_: Biochar from peanut peel
*C*_*0*_: Initial Phosphorus Concentration
CoFe_2_O_4_: Cobalt Ferrite
EDX: Energy Dispersive X-ray
Fe_2_O_3_: Iron Oxide/Ferric Oxide
Fe_3_O_4_: Iron Oxide /Ferrous Ferric Oxide
FTIR: Fourier Transform Infrared
MF: Manganese Ferrite
MFBC: Manganese Ferrite Biochar
MFBC_b_: MnFe_2_O_4_ supported on bagasse
MFBC_p_: MnFe_2_O_4_ supported on peanut peel
MgFe_2_O_4_: Magnesium Ferrite
MnFe_2_O_4_: Manganese Ferrite
MNPs: Magnetic Nanoparticles
NiFe_2_O_4_: Nickel Ferrite
pH: Potential of Hydrogen
SEM: Scanning Electron Microscopy
ZnFe_2_O_4_: Zinc Ferrite
